# Comparison of the external physical damages between laser-assisted and mechanical immobilized human sperm using scanning electronic microscopy

**DOI:** 10.1371/journal.pone.0188504

**Published:** 2017-11-27

**Authors:** David Y. L. Chan, Tin Chiu Li

**Affiliations:** Department of Obstetrics and Gynaecology, The Chinese University of Hong Kong, Hong Kong SAR; Peking University Third Hospital, CHINA

## Abstract

We aim to visualize the external physical damages and distinct external phenotypic effects between mechanical and laser-assisted immobilized human spermatozoa using scanning electronic microscopy (SEM). Human spermatozoa were immobilized mechanically or with laser assistance for SEM examination and the membrane integrities were checked on both types of immobilized spermatozoa. We found evidence of external damages at SEM level on mechanically kinked sperm, but not on laser-assisted immobilized sperm. Although no external damage was found on laser-assist immobilized sperm, there were two distinct types of morphological changes when spermatozoa were stricken by infra-red laser. Coiled tails were immediately formed when Laser pulse was applied to the sperm end piece area, whereas laser applied to the sperm principal piece area resulted in a sharp bend of sperm tails. Sperm immobilized by laser did not exhibit any morphological change if the laser did not hit within the on-screen central target zone or if the laser hit the sperm mid piece or head. Our modified membrane integrity assay revealed that the external membrane of more than half of the laser-assisted immobilized sperm remained intact. In conclusion, mechanical immobilization produced membrane damages whilst laser-assisted immobilization did not result in any external membrane damages besides morphological changes at SEM level.

## Introduction

Intracytoplasmic sperm injection (ICSI) is a powerful technique in Assisted Reproductive Technologies (ARTs) to achieve in vitro fertilization. It can achieve fertilization by bypassing several physical barriers such as the zona pellucida and oolemma, allowing injected sperm to activate the oocyte. This technique is particularly useful in the treatment of severe oligospermic patients whose semen samples cannot be used for conducting conventional IVF. To perform ICSI, immobilization of sperm prior to injection is an essential step for successful fertilization to occur [1]. There are two main benefits of immobilizing sperm before injection: 1.) to prevent movement of the sperm which may damage internal structure of the oocyte; 2.) to permeabilize the sperm membrane in order to release sperm factors to activate the oocyte.

Mechanical immobilization is achieved by slicing across on top of the sperm tail using an ICSI injection needle. This method has been used as a “classical” method for sperm immobilization for nearly 15 years until laser system became an important piece of equipment in ART laboratories. A non-contact diode laser system for sperm immobilization was proposed in 1999 by Schopper *et al*. [2]. Later on, the first trial of laser-assisted ICSI on human oocytes occurred in 2001 [3]; soon followed by the delivery of the first laser-assisted ICSI baby in 2002 [4]. More recently a study by Vizziello *et al*. showed that there were no differences in fertilization rate and cleavage rate between the two immobilization methods [5]. However, the effect of the two different immobilization methods on human sperm morphology has not been examined in detail by scanning electronic microscope. In this study, we aim to employ SEM to compare the impact of the different techniques and methods of immobilization on the external morphology of the sperm.

## Methods and materials

### Semen samples

Semen samples were collected from men who underwent semen analysis as part of investigations for infertility at the IVF unit of Prince of Wales Hospital, Chinese University of Hong Kong. All semen samples were obtained by masturbation after 3–7 days of sexual abstinence and showed normal semen parameters according to the 5^th^ WHO semen analysis guidelines [6].

The data were analyzed anonymously. The authors had access to identifying information. All patients were consented and explained that only left over samples after routine standard semen analyses would be used for this study. Ethics was also obtained from "Joint Chinese University of Hong Kong-New Territories East Cluster Clinical Research Ethics Committee" with the approval reference project No CREC 2016.499. Samples were obtained from our andrology unit between 1st Nov 2016 and 30 April 2017. Routine semen analyses were performed in addition to sperm processing, rapid membrane permeability test and SEM preparation as research procedures.

### Semen analysis

Semen analyses were performed manually according to World Health Organization guidelines (version V) [6]; after complete liquefaction of the samples. Semen volume, pH, sperm concentration, motility, and morphology were measured according to established laboratory protocol. To evaluate sperm morphology, the slides were stained with a Diff-Quik staining kit (Dade Behring AG, Düdingen, Switzerland) and strict criteria were applied to sperm viewed under a Nikon microscope with an oil immersion ×100 objective (Nikon Company, Tokyo Japan). Sperm samples were considered to be ‘normal’ when parameters met or exceeded the WHO reference values: concentration ≥15×10^6^/mL, total motility ≥40%, and morphology ≥4% [7]. Samples with one or more abnormal semen parameters were excluded from this study.

### Sperm processing

All semen samples underwent gradient centrifugation for sperm purification. In brief, 1 ml of 80% and 40% gradient solutions (Irvine scientific) were overlaid in a 15 ml (Falcon) respectively. Then 1 to 2 ml of semen sample was overlaid onto the gradient solution and underwent centrifugation at 1500 rpm for 15 minutes. Supernatants were then removed, and the sperm pellet was washed twice in G-IVF solution with 10% of Serum Substitute Supplement (Irvine Scientific, 99193). Finally, the sperm pellet was resuspended in G-IVF solution to obtain a sperm concentration of around 5 million/ml.

### Sperm manipulation and immobilization

**Fig 1:**
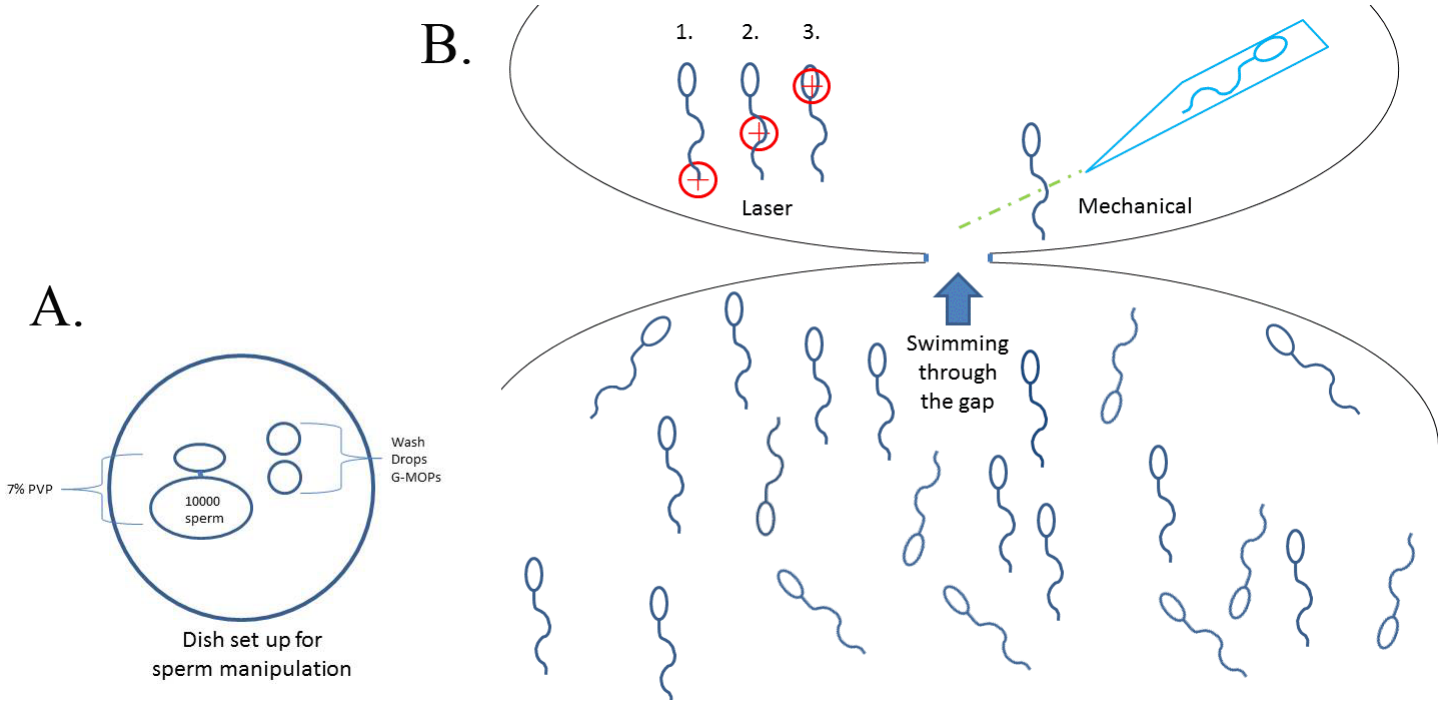
ICSI dish set-up for sperm immobilization. **(A)** In brief, 2 large drops (10 and 15 ul) of 7% PVP were placed very close to each other. A few drops of GMOPs were placed next to the PVP drops. 5 ml of Ovoil (Vitrolife, 10029) was added to cover the drops. A microinjection needle for ICSI was used to slice across the two drops resulting in a narrow and thin channel of PVP between the two PVP drops to allow sperm to swim through. (B) Micromanipulation of sperm, enlarged view of the sperm drop from A. Either laser or mechanical immobilization was performed on swim-across-sperm and the immobilized sperm were picked up by the injection needle. The immobilized sperm were then transferred to wash drops next to the PVP drops for further SEM preparation or membrane integrity test.

ICSI dishes (Nunc) were used for micromanipulation of sperm. 7% PVP (Irvine Scientific, 90121) was used for decelerating sperm movement for easier manipulation. Sperm were then transferred to a GMOPS (G-MOPS, Vitrolife) drop for further SEM preparation. An ICSI injection needle was used for mechanical sperm immobilization and manipulation (Origo, Humagen, MIC-50-35). In mechanical immobilized group, one or two kinks were applied on sperm principle piece only as this is the routine procedure during Intracytoplasmic Sperm Injection (ICSI). In Laser-Assisted immobilized group, a non-contact infra-red laser system (Research Scientific, Saturn 3 active) was used for immobilization. For manipulation procedure, due to the limitation of the laser beam diameter (~7.8μm), the sperm were sub-divided into three target regions for immobilization as: 1) head and midpiece region, 2) principle piece region and 3) end piece region for manipulation procedure. Fig 1 describes the sperm manipulating methods used, dish preparation and micromanipulation of immobilized sperm. At least 300 sperm in each manipulated group were immobilized and at least 3 independent sets of experiments in each group were performed. Sperm after gradient centrifugation and washing steps without any manipulation were used as control sperm.

### SEM preparation

Immobilized sperm in each group and the control group without immobilization were washed with 0.1M pH7.2 Sorensen’ phosphate buffer and then fixed in 2.5% glutaraldehyde fixative for at least 15 minutes on a 13mm filter membrane (SPI Pore PC Track Etc, 0.02 pore size). Sorensen’ phosphate buffer was used to wash the sperm samples three times, each time lasting 5 minutes. Osmium tetroxide was added to the samples for at least 15 minutes. Samples were washed again 3 times in distilled water for 10 minutes each. The samples were then dehydrated in sequential 70–100% concentration of ethanol for 5 minutes each. Samples were dried in a critical point dryer and then loaded onto specimen stubs. Gold-palladium was coated in a Sputter Coater for the final step [8, 9].

### SEM

Sperm samples were then examined under a Hitachi SU8020 SEM Cold Field Emission Microscope (Japan Hitachi). Secondary electron images were taken at low electron energies at 5 keV.

### Rapid (within 5 minutes) membrane permeability test

In order to verify if immobilization did disrupt permeability of the sperm membrane, immobilized sperm were immediately examined for their membrane integrity within 5 minutes. 5g/L eosin was dissolved in saline and then was filtered sterile. After a single pulse of laser or single kink of mechanical immobilization, the sperm sample was then transferred to 0.5μl eosin micro-drop on a glass slide. After a 25mm X 25mm cover slip was applied onto the slide, the sample was checked for membrane permeability within 5 minutes under Olympus BX43 upright microscope. Permeabilized sperm were identified by color in red (stained) in sperm while sperm with intact membrane remained colorless and transparent (unstained). 20 to 30 sperm were immobilized at each manipulation and at least 200 sperm were analyzed.

## Results

### Semen analyses and processing

A total of 5 semen samples were studied, the mean (± SD) age of the men was 32 (±2), the mean (± SD) volume was 3.0 ml (± 0.9), concentration was 50.2 M/ml (± 19.7), motility (PR+NP) was 48% (± 7) and normal morphology according to strict criteria was 5% (± 1). After the sperm processing, the mean (± SD) motility was 93% (± 2).

### Scanning electronic microscope

**Fig 2:**
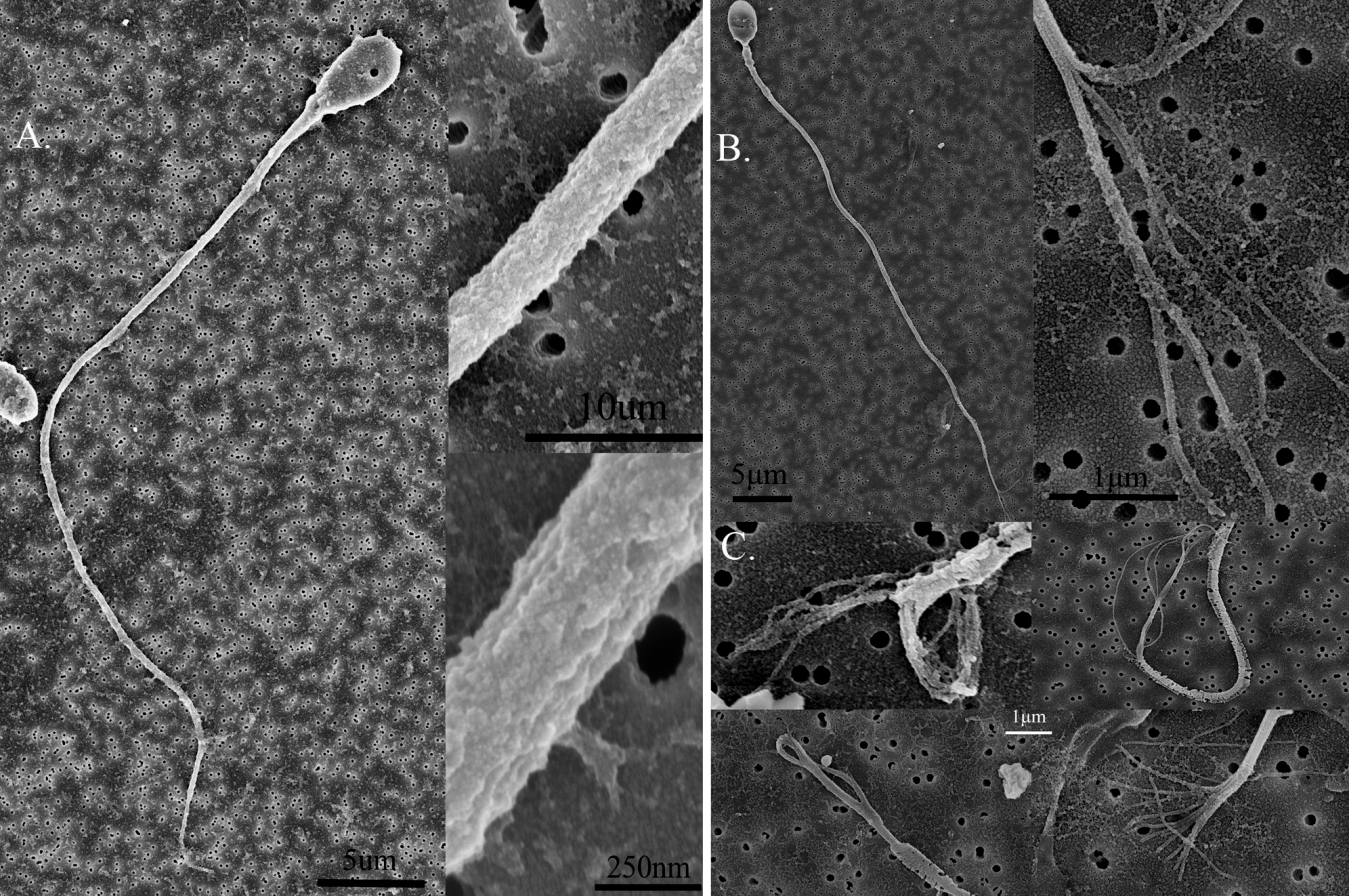
Non-immobilization control group. (A) Non-immobilized spermatozoa showed intact external structure without any damage. Under 40,000X magnification, the whole sperm structure was clearly seen and the tail piece was smooth and intact. (B) Interestingly, Around 5% of sperm in this group showed degradation of the end piece and release of the axoneme. (C) The different extents of denuded end pieces. Background is filter membrane with 0.22 μm pore size.

In the control group, we did not find any external damage on the principle piece of non-immobilized sperm (Fig 2A), but around 5% of sperm (11/206) in the control group displayed end piece membrane degradation and the release of the denuded axoneme to different extents (Fig 2B and 2C).

**Fig 3:**
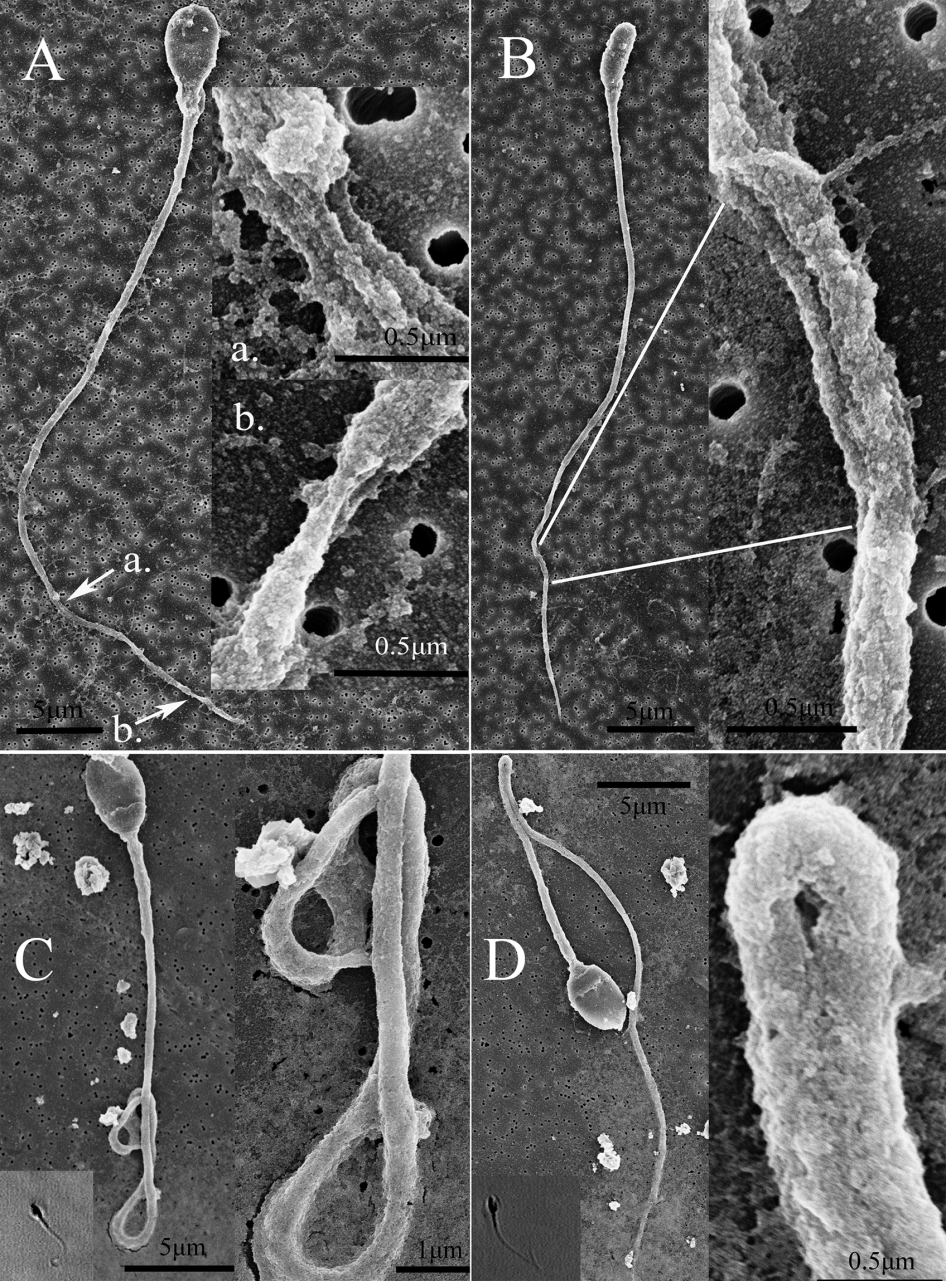
Mechanical and laser immobilization groups. In mechanical immobilization group (3A and B), two kinks were applied onto the “A” spermatozoon. The tail circumference was reduced around the kinked area and sperm tail external structure was damaged by the action. Internal axoneme structures were seen under high magnifications. The intracellular matrix seemed to be released from the damaged tail lesion and was fixed alongside with the sperm during preparation. A single kink was applied on the “B” spermatozoon. The tail was twisted and the damaged area was opened up by mechanical immobilization. In laser-assisted immobilization group (3C and D), laser fired on the end piece area resulted in a coiled tail (C) and, when the laser hit the principle piece area, an abrupt bending of the principle piece resulted (D). Under high magnification SEM showed no external membrane damage compared to mechanical immobilization. Inlets in C and D showed similar sperm morphology after laser immobilization *in vitro* under 400x magnification. The background holes were 0.22μm pore size on a filter membrane.

In the mechanically immobilized group, one or two kinks were applied onto each sperm. We found sperm membranes were torn opened and/or axoneme-like internal structures were exposed. A number of sperm were twisted at the principle piece region. The external damages on the principle piece of sperm were clearly seen (Fig 3A and 3B).

In the laser-assisted immobilization group, there were several patterns of morphological changes observed. 1.) No morphological change; 2.) Completely bent tail (Fig 3D); 3.) Coiled tail formation (Fig 3C). The frequency of these 3 different types of morphological changes depended on which part of the sperm was hit by the laser (Table 1). When the laser hit the principal piece, the predominant pattern was no morphological change; only around 8% of sperm showed a sharp bent tail. In contrast, if the laser hit the end piece area, 94% formed coiled tail, whereas if the laser hit the head or neck area then no morphological change was observed at all. Regardless of the morphological changes, all sperm were successfully immobilized by only one laser pulse and SEM did not find any external damage in this group.

**Table 1:** Frequency of morphological changes by LAI.

	LAI regions		
	Sperm tail end piece	Principle piece	Head and Mid piece
Bended Principle Piece	0 (0%)	15 (8%)	0 (0%)
Coiled tail	189 (94%)	0(0%)	0(0%)
No change	11 (6%)	205 (92%)	200 (100%)
Total Number	201	220	200

Comparison of the frequency of the different morphological changes observed following immobilization by applying laser on various parts of the sperm.

### Rapid (within 5 minutes) sperm membrane permeability test

The rapid membrane integrity test showed that up to 95% of mechanically kinked sperm lost membrane integrity within 5 minutes, while more than half (55%) of the laser assisted immobilized sperm retained membrane integrity during the same five minute period (Table 2). We performed Chi-square analysis that showed significant difference between groups. Our results showed that even though both of the immobilization methods successfully stopped the sperm movement, the laser pulse did not deprive the majority of sperm membrane integrities immediately. Thus, more than half of the sperm retained membrane potential. Table 2 compares the results of the membrane integrity test between the two immobilization methods.

**Table 2:** Rapid sperm membrane permeability test.

	Mechanical	Laser Assisted	*p*-value (Chi Square test)
Live sperm (%)	11 (5%)	125 (55%)	<0.00001
Dead sperm (%)	205 (95%)	101 (45%)	<0.00001
Total immobilized sperm	216 (100%)	222 (100%)	

Comparison of the results of membrane permeability test between the two immobilization methods. 95% of mechanically immobilized sperm lost membrane integrity within 5 minutes. Meanwhile, less than 5% of sperm retained membrane integrity. In contrast, 45% of laser assisted immobilized sperm lost membrane integrity, however, 55% of sperm retained permeability to a significant level (>0.00001) compared with the mechanically immobilized live sperm (5%) group.

## Discussion

To the best of our knowledge, this is the first study ever conducted to compare the impact of the two different types of immobilization methods on human sperm morphology and function. We observed external damages from mechanically immobilized sperm but not laser-assisted immobilized sperm at the SEM level. Since our SEM revealed no external damage on laser-assisted immobilized sperm, we speculated that the membrane integrity might be preferentially preserved. Therefore, we subsequently conducted a modified membrane integrity test in order to determine the membrane potential. The test showed that 55% of laser-assisted immobilized sperm retained membrane integrity, while only 5% of mechanically immobilized sperm were still alive; these results demonstrated that laser-assisted immobilization imposed less damage to the sperm membrane compared to the mechanical method. This study is also the first to look at morphological responses to a laser-assisted immobilization method in region specific manner. Our observation showed differential morphological changes at different sperm regions after the laser immobilization.

In the literature, there have been only two studies on the effect of mechanical immobilization on the ultrastructure of the human sperm. Takeuchi *et al*. reported a case of membrane disruption of the head region demonstrated by Transmitted Electronic Microscopy following mechanical immobilization [10]. In contrast, another report by Gomez-Torres *et al*. observed SEM evidence of structural damage in the tail and neck region [11]. However, the frequency in which these structural damages occur following mechanical immobilization is not known. In this study, every SEM examined sperm from the mechanical immobilization group showed membrane impairment, whereas none of the laser immobilized sperm showed external damage. The results visualized that the laser inactivated the sperm movement via a different mechanism. To date, there is no complete explanation on how the laser can impair sperm motility. On the other hand, there is no SEM study so far to look at laser-assisted immobilized sperm; and it is also unclear whether the change in frequency of the membrane potential is higher or lower in laser-assisted immobilization. Our modified viability test first showed that more than half of the laser immobilized sperm retained membrane potential within 5 minutes, whereas 95% of mechanically immobilized sperm lost membrane integrity within the same time interval. Our data showed a significant difference in membrane integrity between the two immobilization methods. Our results also raise an interesting question that how can laser immobilized sperm trigger the activation of the oocyte without losing membrane integrity? It is believed that losing membrane integrity to release sperm activation factors is critical for fertilization to occur, so it will be interesting to examine how sperm activates fertilization without losing membrane integrity. Another possible explanation is that the laser power may create cracks on the membrane at a nano-scale that is out of SEM detectable range.

In addition, the possible impact of laser on different parts of the sperm and the resulting morphological changes has not been previously reported. Our results showed that 94% of sperm showed coiling over the end piece if the laser was directed to this part, in contrast to only 8% of sperm showing a sharp bend in the principal piece if the laser was directed to this region. We observed that laser assisted immobilization is the only immobilization method which can produce coiling or sharp bend on human sperm. Our study not only examined the morphological changes when using both mechanical and laser assisted immobilization, but also the sperm membrane integrity. Interestingly, in our non-immobilized group, we found around 5% of sperm showed a denuded terminal piece, which indicated that this phenomenon may be a process of natural degradation rather than post-manipulation damage reported by Gomez-Torres et al. However, this phenomenon needs further investigation to give a conclusive result.

There were two limitations in this study. For the SEM observation, our limitation was not being able to recover all immobilized sperm. Losing sperm was inevitable during SEM sample preparation procedures. In order to overcome this situation, hundreds of sperm needed to be manipulated in each group and only a few dozens of sperm were retained for SEM examination. The other limitation of this study was that we were not able to measure sperm membrane permeability in real time. However, we strived to measure membrane integrity within 5 minutes. It was technically challenging to count less than 30 sperm in a 10mm X 10mm area within 5 minutes. Our modified membrane integrity test was also a novel way to show this new finding that there were membrane integrity discrepancies between the two immobilization methods.

Mechanical immobilization is the immobilization method most typically used in ART clinics worldwide. The technique is easy to perform with no additional piece of equipment required. Although laser-assisted immobilization was first introduced into the field more than ten years ago, it had been shown in only one study that its efficacy is equivalent to that of the mechanical immobilization method causing no DNA damage [12]. However, its popularity and utilization is still rather limited, partly because of increased cost and partly because increased manipulation time which together appear to have hindered the widespread use of laser-assisted immobilization in ART clinics.

It is interesting to know whether different immobilization methods will impose different timings of fertilization due to the membrane integrity discrepancy. We speculate that since the mechanical method permeabilizes the sperm membrane faster than the laser assisted method, this may lead to a shorter fertilization time because the sooner the sperm activating factors are released from the sperm lesions, the sooner they may trigger oocyte activation. It will also be interesting to investigate with a large sample size to see whether different sperm immobilization methods will give different patterns of embryogenesis outcomes. Only one study [13] reported that there was no difference in the efficacies between the two immobilization methods. To our knowledge, there is no large scale RCT comparing the two immobilization methods and their effects on fertilization rates, cleavage rates, blastulation rates, pregnancy rates and live birth rates. Such a cohort RCT study will give a better insight into an improved immobilization method and protocol development for ART clinics worldwide.

## Conclusion

Mechanical and laser assisted immobilization are the only two ways to stop sperm movement for ICSI, however the external damages and membrane integrity that result from the two methods have never been visualized in detail. Our images revealed the impacts on morphological changes and membrane function of sperm after using the two methods. In summary, the laser-assisted immobilization group showed distinctive morphological changes without observable external damage on the sperm at SEM level compared to the mechanical group. There is a lack of large scale data to support whether laser assisted immobilization is as good as the mechanical method, thus it is interesting to know the discrepancies of the physical damages between the two different immobilization methods. Future studies may focus on the effect of these different immobilization methods on the subsequent development of ICSI-derived zygotes or embryos.
